# Day 15 and Day 33 Minimal Residual Disease Assessment for Acute Lymphoblastic Leukemia Patients Treated According to the BFM ALL IC 2009 Protocol: Single-Center Experience of 133 Cases

**DOI:** 10.3389/fonc.2020.00923

**Published:** 2020-06-30

**Authors:** Letitia-Elena Radu, Andrei Colita, Sergiu Pasca, Ciprian Tomuleasa, Codruta Popa, Catalin Serban, Anca Gheorghe, Andreea Serbanica, Cristina Jercan, Andra Marcu, Ana Bica, Patric Teodorescu, Catalin Constantinescu, Bobe Petrushev, Minodora Asan, Cerasela Jardan, Mihaela Dragomir, Alina Tanase, Anca Colita

**Affiliations:** ^1^Department of Medicine, Carol Davila University of Medicine and Pharmacy, Bucharest, Romania; ^2^Department of Stem Cell Transplantation, Fundeni Clinical Institute, Bucharest, Romania; ^3^Department of Hematology, Coltea Hospital, Bucharest, Romania; ^4^Department of Hematology, Iuliu Hatieganu University of Medicine and Pharmacy, Cluj Napoca, Romania; ^5^Research Center for Functional Genomics and Translational Medicine, Iuliu Hatieganu University of Medicine and Pharmacy, Cluj Napoca, Romania; ^6^Department of Hematology, Oncology Institute Prof. Dr. Ion Chiricuta, Cluj Napoca, Romania; ^7^Medfuture Research Center for Advanced Medicine, Iuliu Hatieganu University of Medicine and Pharmacy, Cluj Napoca, Romania

**Keywords:** children, ALL, MRD, flow cytometry, survival

## Abstract

**Introduction:** Childhood acute lymphoblastic leukemia (ALL) is a hematologic malignancy characterized by the acquisition of several genetic lesions in the lymphoid progenitors with subsequent proliferation advantage and lack of maturation. Along the years, it has been repeatedly shown that minimal residual disease (MRD) plays an important role in prognosis and therapy choice. The aim of the current study was to determine the prognostic role of MRD in childhood ALL patients in conjunction with other relevant patient and disease characteristics, thus showing the real-life scenario of childhood ALL.

**Patients and Methods:** The retrospective study includes childhood ALL patients that were treated according to the BFM ALL IC 2009 between January 2016 and December 2018 at the Fundeni Clinical Institute, Bucharest, Romania.

**Results:** None of the variables significantly influenced the induction-related death in our study. None of the variables independently predicted relapse-free survival (RFS) with the highest tendency for statistical significance being represented by poor prednisone response. Non-relapse mortality (NRM) was independently predicted by age, prednisone response, and day 33 flow cytometry-MRD (FCM-MRD). Overall survival (OS) was independently predicted by prednisone response and day 33 FCM-MRD. Event-free survival (EFS) was independently predicted by age, prednisone response, and day 33 FCM-MRD.

**Conclusion :** Prednisone response, day 15 FCM-MRD, day 33 FCM-MRD, and the risk group represent the most important factors that in the current study independently predict childhood ALL prognosis.

## Introduction

Acute lymphoblastic leukemia (ALL) is the most common malignancy in children, responsible for 30% of all pediatric neoplasms (1). Childhood ALL is characterized by the acquisition of several genetic lesions in the lymphoid progenitors with subsequent proliferation advantage and lack of maturation. The clinical presentation of this condition is generally the result of bone marrow infiltration by lymphoid blasts and involvement of extramedullary organs (2).

Childhood ALL mortality has decreased since the 1970s (3) with some patient categories reaching cure rates of 90% as a direct consequence of better patient stratification and therapeutic approach (4). One of the most important variables in patient stratification is minimal residual disease (MRD) assessment and risk stratification (5). Initially, MRD was assessed by a few study groups, but currently most ALL protocols include MRD evaluation (6). The sensitivity of MRD detection ranges according to the technique used to detect it. This ranges from the flow cytometry MRD (FCM-MRD) detection in the bone marrow followed by more sensitive methods, polymerase chain reaction (PCR) for fusion genes or immunoglobulin/T-cell receptor gene rearrangements and next-generation sequencing (NGS). Still, some molecular techniques are more difficult to implement in the clinical scenario because of lack of standardization, costs, or absence of a specific target (6, 7). It has been shown that MRD assessment has a big impact not only in determining the prognosis of ALL patients, but also in tailoring the therapeutic management, this strategy being used by multiple ALL protocols (8–11). Nevertheless, MRD might not be sufficient in all subtypes of patients with the need of assessing additional disease characteristics as is the case of cytogenetic risk (12, 13).

Relapse-free survival (RFS) and overall survival (OS) are influenced by the disease itself and by the complications during therapy. Despite the progress in treating this disease, ~20% of patients still relapse (14). Thus, the aim of the current study is to determine the results of BFM ALL IC 2009 in our clinical center, showing a real-life scenario of childhood ALL.

## Patients and Methods

### Patients

This is a retrospective study that included all newly diagnosed ALL patients between January 2016 and December 2018 in the Department of Hematology and Stem Cell Transplantation, Fundeni Clinical Institute, Bucharest, Romania, with follow-up until December 2019. Children under the age of 1 and with L3 morphology or bilineal/biphenotype ALL were excluded. The legal guardians signed an informed consent prior to the enrollment. The study was in accordance with the declaration of Helsinki and received approval from the ethical committee from the Fundeni Clinical Institute, Bucharest, Romania (6323/04.02.2020). Therapy followed the BFM ALL IC 2009 treatment plan.

### Study Definitions

Severe anemia was defined as hemoglobin under 7 g/dL. Severe thrombocytopenia was defined as platelets under 50 × 10^9^/L.

Study variables included gender, age at diagnosis, environment, white blood cells, blasts, hemoglobin and platelet counts in peripheral blood smears, lymphoblast morphology, immunophenotype and cytogenetics from bone marrow aspirate at diagnosis, molecular biology (for TEL-AML1/ETV6-RUNX1, BCR-ABL1 p190 and p210, SIL-TAL1, E2A-PBX1/TCF-PBX1, MLL-AF4/KMT2A-AFF1), initial central nervous system involvement, prednisone response, and risk stratification as well as MRD analysis on days 15, 33, and 78.

MRD measurement was performed using a 10-color flow cytometry analyzer (Navios, Beckman Coulter). We identified abnormal expression of immunophenotypic markers defined as leukemia-associated aberrant immunophenotype, using a combination of eight markers and reaching 10^−4^ level of sensitivity.

Risk groups were defined based on BFM ALL IC 2009 protocol: standard risk group (SRG): < 1 × 10^9^/L blasts on peripheral blood smear on day 8, age between 1 and 6 years, initial leukocyte count < 20 × 10^9^/L, MRD on day 15 < 0.1%, and < 5% blasts on bone marrow aspirate on day 33; high-risk group (HRG): hypodiploidy or *t*_(9;22)_ or *t*_(4;11)_ or more than 1 × 10^9^/L blasts on peripheral blood smear on day 8 or MRD on day 15 >10% or more than 5% blasts on bone marrow aspirate on day 33; intermediate-risk group (IRG): patients not stratified as SRG or HRG.

Prednisone response was defined as day 8 absolute blast count under 1000/μL. It must be mentioned that day 8 was preceded by 7 days of prednisone and one dose of intrathecal methotrexate on day 1. Bone marrow status was defined as M1 if <5% blasts were found on bone marrow aspirate, M2 ≥5% and <25% blasts, M3 ≥25%. Day 15 FCM-MRD groups were defined as under 0.1%, 0.1%−1%, 1%−10%, 10% or more. The previously mentioned intervals are opened to the right and closed to the right. Day 33 FCM-MRD groups were defined as under 0.05 and 0.05% or more.

We defined relapse-free survival (RFS) as interval of time from diagnosis to relapse of any kind. We defined NRM as the interval of time from diagnosis to death of patients that did not relapse; in this case, the time of relapse was right censored.

Causes of death were documented: death in induction, treatment, and non-treatment-related mortality. We defined OS as the interval time from diagnosis to death of any cause. Induction-related death was defined as death before day 33. Event-free survival (EFS) was defined as the interval of time from diagnosis to either death or relapse.

### Data Analysis

Data analysis was performed using R 3.5.3. Categorical variables were represented as absolute value (percentage). Contingency tables were analyzed using the Fisher test. The Shapiro test and histogram visualization were used to assess the normality of the distribution. Normally distributed variables were represented as mean ± standard deviation, and non-normally distributed variables were represented as median (quartile 1, quartile 3). Differences between two normally distributed groups were assessed using the *t*-test. Differences between two non-normally distributed groups were assessed using the Mann–Whitney–Wilcoxon test. Univariate survival analysis was performed using a univariate Cox proportional hazards model. Variables that reached a *p*-value under 0.1 in the univariate Cox proportional hazards model were further used in the multivariate Cox proportional hazards model with the exception of the case in which both morphologic bone marrow involvement and FCM-MRD of the same day reached the inclusion criteria, in which case we selected only the FCM-MRD, considering their known association. If both day 15 and day 33 FCM-MRD reached the inclusion criteria, the day with the lowest *p*-value was included in the multivariate analysis. The risk group was not included in the multivariate analysis considering that it is composed of other variables that would be included in the multivariate model. In the case in which there was no event in one of the selected groups and the Cox proportional hazards model was not suitable, we used the log-rank test to generate a *p*-value and interpreted the Kaplan–Meyer curves to determine the direction of the effect. A *p*-value under 0.05 was considered statistically significant.

## Results

We have included 133 childhood ALL patients in the current study with the general characteristics presented in Table 1. The median follow-up of the cohort was of 810 (490, 1076) days. None of the 103 (77.44%) patients evaluated at day 78 presented MRD positivity.

**Table 1:** General characteristics of the cohort.

			***n* = 133**
Sex	Female		53 (39.85%)
	Male		80 (60.15%)
Area	Rural		59 (44.36%)
	Urban		74 (55.64%)
Age (years)			5 (2, 10)
Age groups	1–6 years		80 (60.2%)
	7–10 years		22 (16.5%)
	11–17 years		31 (23.3%)
Leukocytes × 10^9^/L			11.1 (4.92, 32.77)
Leukocyte groups	<10 × 10^9^/L		61 (45.9)
	10–20 × 10^9^/L		21 (15.8)
	20–50 × 10^9^/L		25 (18.8)
	>50 × 10^9^/L		26 (19.5)
Hemoglobin (g/dL)			8.74 +/– 2.4
Hemoglobin groups	<7 g/dL		30 (22.56%)
	7–10 g/dL		71 (53.38%)
	>10 g/dL		32 (24.06%)
Platelets × 10^9^/L			51 (29, 112)
Platelet groups	<20 × 10^9^/L		15 (11.28%)
	20–50 × 10^9^/L		49 (36.84%)
	50–100 × 10^9^/L		32 (24.06%)
	>100 × 10^9^/L		37 (27.81%)
Morphology	L1		129 (97%)
	L2		4 (3%)
Immunophenotype	B	proB	2 (1.5%)
		B common	84 (63.16%)
		preB	25 (18.79%)
	T		22 (16.54%)
Cytogenetics	No evaluable metaphases		40 (30%)
	Normal karyotype		46 (34.6%)
	Abnormalities		47 (35.4%)
Molecular biology	None		94 (70.8%)
	TEL-AML1/ETV6-RUNX1		24 (18%)
	E2A-PBX1/ TCF3-PBX1		4 (3%)
	MLL-AF4/ KMT2A-AFF1		4 (3%)
	BCR-ABL1 p190		4 (3%)
	BCR-ABL1 p210		2 (1.5%)
	SIL-TAL1		1 (0.7%)
CNS involvement			1 (0.75%)
Prednisone response	Good		115 (86.47%)
	Poor		18 (13.53%)
Risk group	High		44 (35.2%)
	Intermediate		61 (48.8%)
	Standard		20 (16%)
Day 15 bone marrow morphologic disease	M1		53 (59.55%)
	M2		27 (21.6%)
	M3		9 (7.2%)
Day 33 bone marrow morphologic disease	M1		109 (99.09%)
	M2		0 (0%)
	M3		1 (0.9%)
Day 15 FCM-MRD groups	<0.1%		29 (25.44%)
	0.1–1%		23 (20.18%)
	1–10%		39 (34.21%)
	>10%		23 (20.18%)
Day 33 FCM-MRD groups	<0.05%		94 (83.93%)
	>0.05%		18 (16.07%)
Day 78 MRD	Positive (>0.01%)		0 (0%)
	Negative (≤0.01%)		103 (100%)

**Figure 1:**
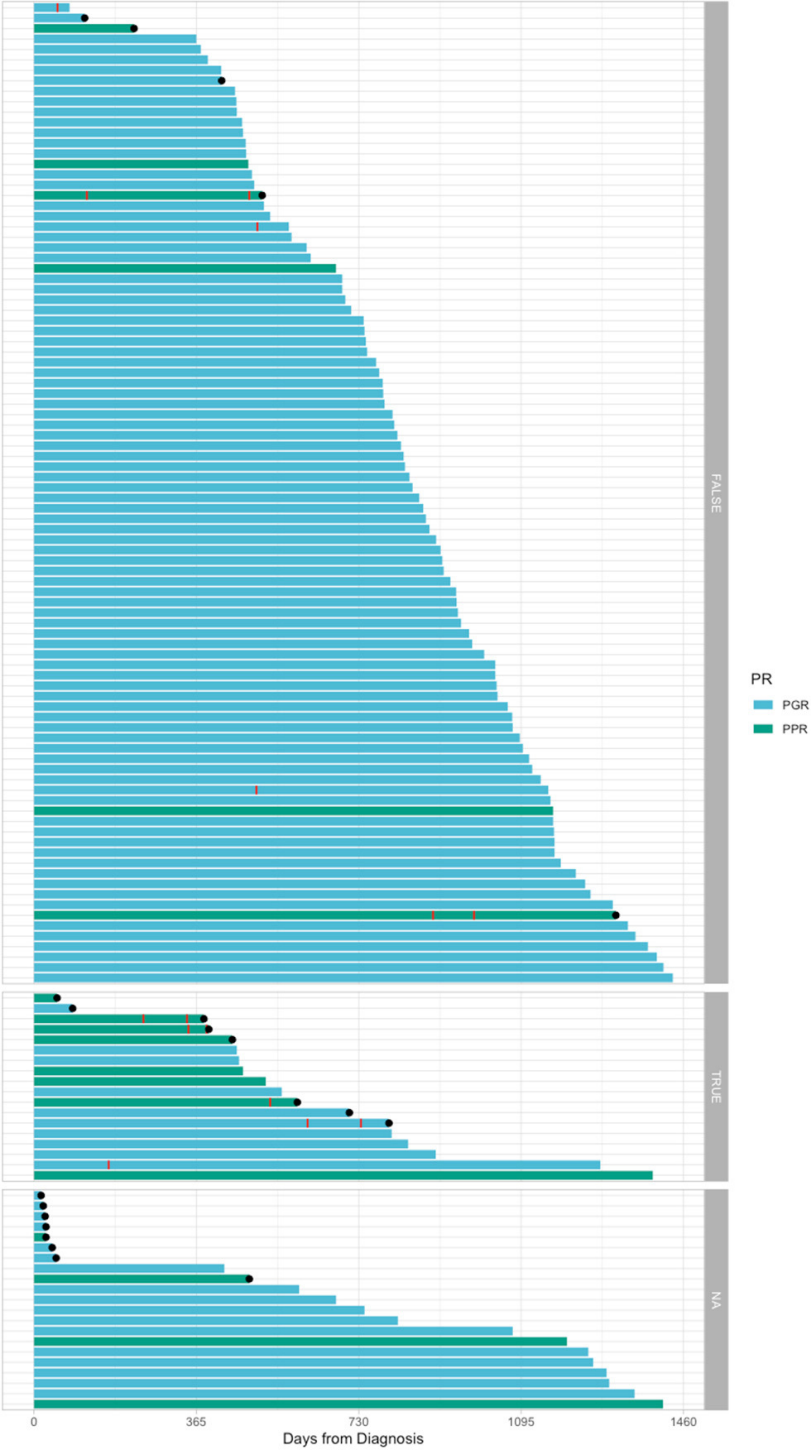
Swimmer plot offering an overview of the cohort. Red stripes represent relapse. Black dots represent death. The plots have been divided according to the bone marrow flow cytometry evaluation at day 33: TRUE = 0.05% ALL blasts or over; FALSE = under 0.05 ALL blasts; NA, no information available. PR, prednisone response; PPR, prednisone poor response; PGR, prednisone good response.

Figure 1 shows an overview of the cohorts and the main endpoints.

**Figure 2:**
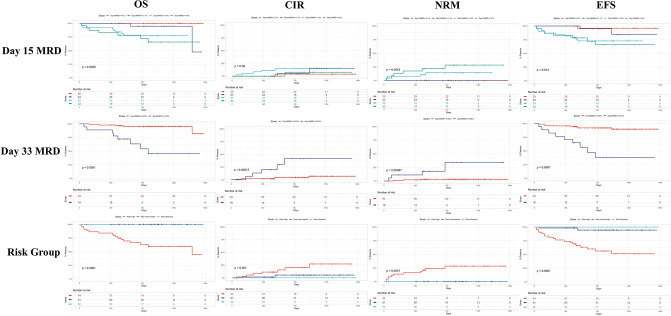
Kaplan–Meyer curves on the influence of the day 15 FCM-MRD, day 33 FCM-MRD, and risk group on OS, RFS, and NRM. As we observed on Kaplan–Meyer curves, for day 15 FCM-MRD the curves of groups under 0.1% and between 0.1 and 1% highly overlap, and so do the curves between 1% and 10% and 10% or more (for OS and EFS). Thus, this represents the reason for dichotomizing the analysis using day FCM-MRD of 1% as the cutoff. CIR = cumulative incidence of relapse.

It can be observed on the Kaplan–Meyer curves from Figure 2 that, for day 15 FCM-MRD, the curves of groups under 0.1% and between 0.1 and 1% highly overlap, and so do the curves between 1% and 10% and 10% or more (for OS and EFS). Thus, this represents the reason for dichotomizing the analysis using day FCM-MRD of 1% as the cutoff. Probably because of the small number of cases that died before day 33, none of the variables showed association with induction-related death (Supplementary Table 1).

In the RFS univariate analysis, there was a negative impact of older age, T-ALL compared to B-ALL, leukocytes over 100 × 10^9^/L, poor prednisone response, high risk group, day 33 morphologic presence of disease, and FCM-MRD over 0.05% (Supplementary Table 2). In the multivariate analysis, none of the included variables were shown to be independently associated with relapse (Supplementary Table 3).

In the NRM univariate analysis, the following variables presented a negative impact: female sex, poor prednisone response, high risk group, day 15 M3 bone marrow, day 33 morphologic presence of disease, and day 33 FCM-MRD over 0.05% (Supplementary Table 4). In the multivariate analysis, older age became a risk factor, and poor prednisone response and day 33 FCM-MRD over 0.05% maintained a poor prognosis association (Supplementary Table 5). There was no association found between day 15 or day 33 FCM-MRD and either hemoglobin or platelet count at diagnosis.

In the OS univariate analysis, there was a negative impact of female sex, poor prednisone response, high risk group, day 15 M3 bone marrow, day 15 FCM-MRD over 1%, day 33 morphological disease, day 33 FCM-MRD over 0.05% (Supplementary Table 6). In the multivariate analysis poor prednisone response and day 33 FCM-MRD over 0.05% remained associated with poor OS (Supplementary Table 7).

In the EFS univariate analysis, older age, severe thrombocytopenia, poor prednisone response, high risk group, M3 morphology on day 15, day 15 FCM-MRD over 1%, day 33 bone marrow morphologic disease, and day 33 FCM-MRD over 0.05% presented a negative impact on EFS (Supplementary Table 8). In the multivariate analysis, older age, poor prednisone response and day 33 FCM-MRD over 0.05% presented independent association with a worse EFS (Supplementary Table 9).

**Figure 3:**
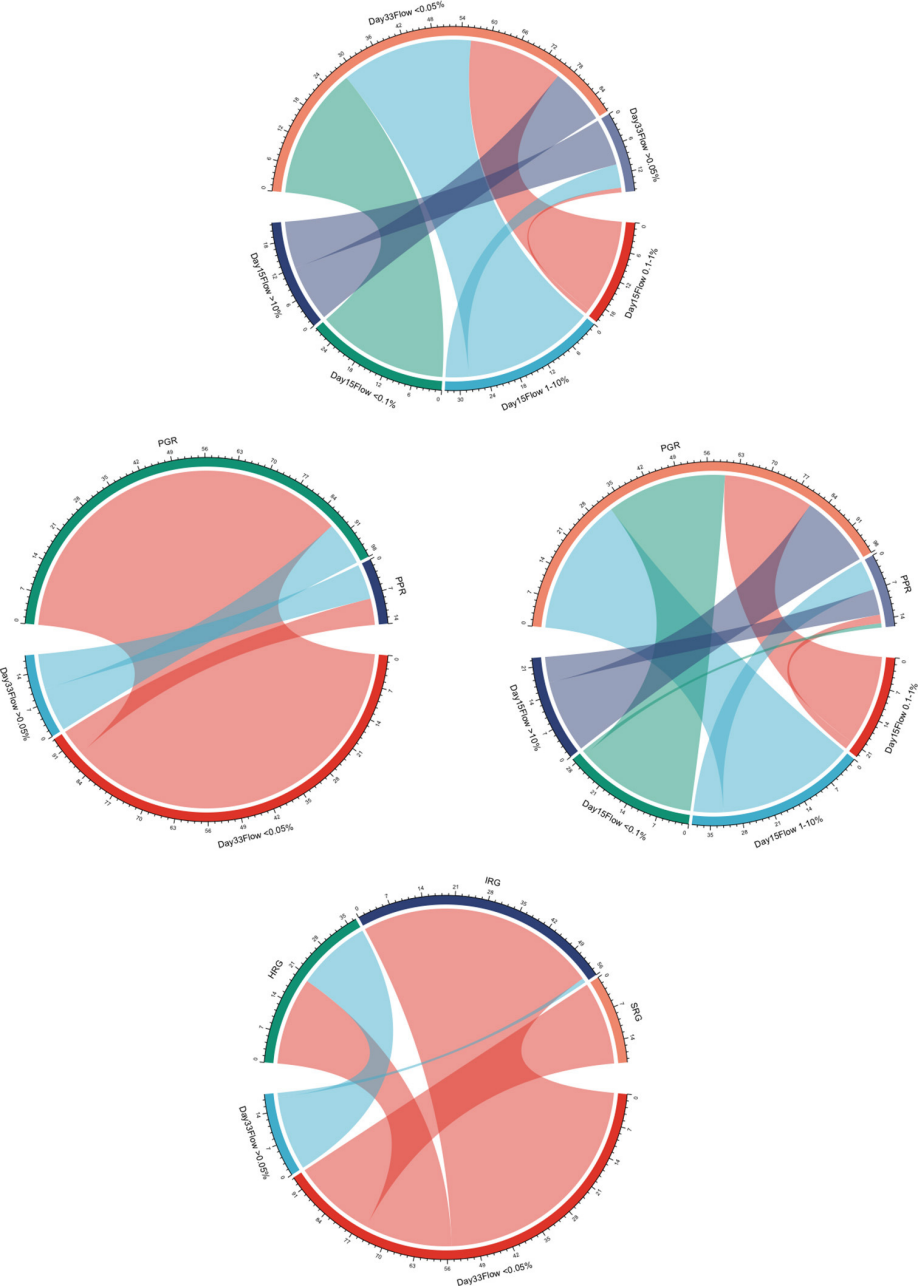
Chord diagrams showing the associations between day 15 FCM-MRD groups, day 33 FCM-MRD groups, risk group, and prednisone response. PPR, prednisone poor response; PGR, prednisone good response; SRG, standard risk group; IRG, intermediate risk group; HRG, high risk group.

We further assessed the association between the factors that presented as statistically significant in the multivariate analysis (Figure 3).

**Figure 4:**
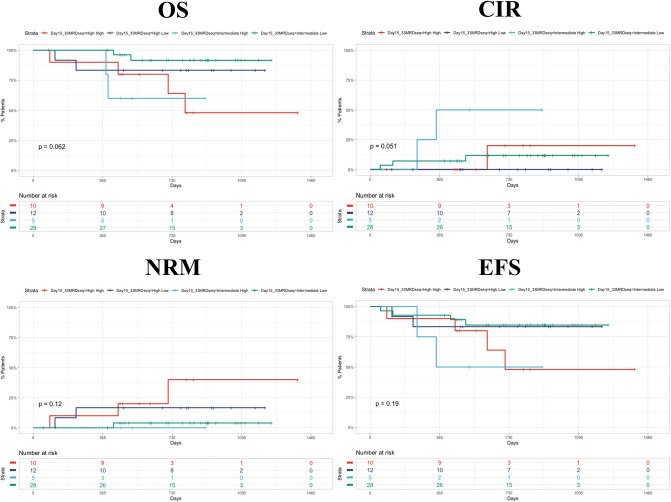
Kaplan–Meyer curves showing the dynamics of day 15 and day 33 MRD. The first word represents the day 15 MRD value (intermediate = 1%−10%; high = 10%−100%). The second word represents day 33 MRD value (low = <0.05%; high = >0.05%). CIR, cumulative incidence of relapse.

In Figure 2, we presented Kaplan–Meyer curves on the influence of the day 15 FCM-MRD, day 33 FCM-MRD, and risk group on OS, RFS, and NRM. Considering that some of the patients presenting day 15 FCM-MRD of over 1% can transition to either day 33 FCM-MRD over or under 0.05% (Figure 3), whereas almost all patients with day 15 FCM-MRD under 1% tend to reach a day 33 FCM-MRD under 0.05, we decided to observe which day has the biggest influence in the subgroup of patients with day 15 FCM-MRD over 1% (Figure 4). Moreover, the importance of the sequential prednisone response followed either by day 15 FCM-MRD over 1% (Supplementary Figure 1) or day 33 FCM-MRD (Supplementary Figure 2) is assessed.

**Figure 5:**
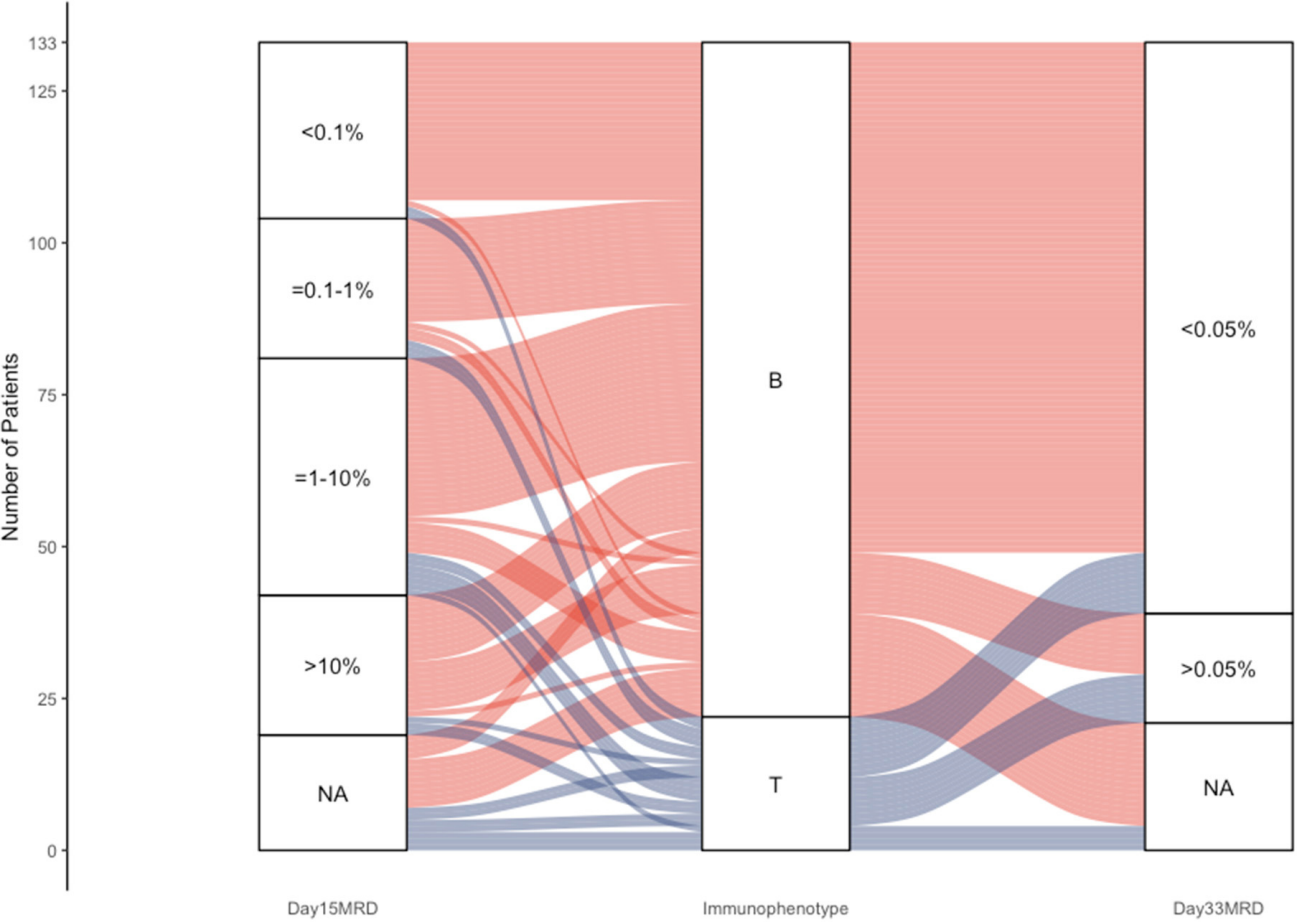
Sankey plot representing the association between immunophenotype and day 15 and day 33 MRD.

In Figure 5, we presented a Sankey plot showing the association between the immunophenotype and day 15 and day 33 FCM-MRD. There was no association between immunophenotype and day 15 MRD (*p* = 0.668), but there was a statistically significant association between immunophenotype and day 33 MRD (*p* = 0.00159). It must be mentioned that in Supplementary Tables 2, 3 it can be observed that immunophenotype and day 33 FCM-MRD predict relapse, but both lose statistical significance in the multivariate analysis. This being said, the *p*-value for the day 33 FCM-MRD is lower than the one for immunophenotype in both the univariate and multivariate analyses, showing that day 33 FCM-MRD is, in the very least, able to replace immunophenotype regarding relapse, thus, making day 33 FCM-MRD relevant regardless of immunophenotype.

## Discussions

The research shows that day 33 FCM-MRD as well as the status of poor prednisone response are among the most important independent factors in predicting OS, NRM, and EFS. The results presented here are in accordance to the vast majority of the literature, showing the high prognostic impact of FCM-MRD (8–11). Nonetheless, this data does not adjust subgroups to better tailor the impact of MRD, but this might also be caused by the relatively low cohort and the low numbers of some types of disease like early T precursor ALL (ETP-ALL) (12, 13).

Although prednisone response was introduced a long time ago, it still has prognostic relevance in the multivariate model (15, 16). Regarding prednisone response, only 18 patients were poor responders, and thus, we could not assess all cases with a bad prognosis.

As mentioned before, the risk groups were not included in the multivariate analysis considering they are assigned in dependence on other factors included. Nonetheless, the prognostic stratification of the risk groups was similar to the presented literature with SRG and IRG Kaplan–Meyer curves being close together and HRG having a highly worse outcome.

T-ALL presented a higher risk of relapsing compared to B-ALL in the univariate analysis—a fact that is in accordance with the known published literature (17, 18). Nonetheless, this is not kept in the multivariate model, showing that, in our experience, FCM-MRD and prednisone can overrule the immunophenotype. Immunological classification is not to be removed from the clinical management considering the different biology of these diseases and, thus, different therapeutic management (17, 18).

Although female patients generally have a better outcome when compared to male patients (19), in the current study, we have observed that, in the univariate analysis, males had a favorable OS and NRM, impact that was not kept in the multivariate analysis. Also, induction-related death was not predicted by any variable, probably because of the low number of events included in this category.

Although not statistically significant, it appears that day 33 FCM-MRD over 0.05% after a day 15 FCM-MRD >1% predicts a worse OS and RFS although this must be validated in larger cohorts. Moreover, we observed that prednisone poor response worsens the prognostic of a patient with day 15 FCM-MRD over 1%. Still, because of the low number of patients included in this subanalysis, there were no patients with day 15 FCM-MRD over 10% and poor prednisone response that relapsed at follow-up. For prednisone response and day 33 FCM-MRD, we report that the association between prednisone good response and FCM-MRD under 0.05% offers the best prognosis, the combination between prednisone poor response and day 33 FCM-MRD over 0.05 has the worst prognosis, and the curves generated by using the other two intermediate combinations offer intermediate survival curves that seemingly overlap.

Interestingly, FCM-MRD was strongly correlated with NRM. NRM is high among patients with high levels of MRD. This subclass of patients underwent intensive chemotherapy with aggressive protocols, thus being severely immunocompromised and cytopenic. The early mortality for these patients is due to cerebral hemorrhage due to severe thrombocytopenia (in two patients) and sepsis (due to therapy-related infections in the rest).

## Conclusions

In conclusion, prednisone response, day 15 FCM-MRD, day 33 FCM-MRD, and the risk group represent the most important factors that independently predict childhood ALL prognosis in the current study.

## Data Availability Statement

The raw data supporting the conclusions of this article will be made available by the authors, without undue reservation.

## Ethics Statement

The studies involving human participants were reviewed and approved by ethical committee from the Fundeni Clinical Institute, Romania. The patients/participants provided their written informed consent to participate in this study.

## Author Contributions

L-ER, SP, and AndC contributed equally to the current manuscript and are all considered first author, having written the manuscript. All the other authors contributed to clinical data collection. AncC supervised the work.

## Conflict of Interest

The authors declare that the research was conducted in the absence of any commercial or financial relationships that could be construed as a potential conflict of interest.

## Supplementary Material

Supplementary Figure 1 Kaplan-Meyer curves showing the dynamics of prednisone response and day 15 FCM-MRD. The first word represents the prednisone response (PGR, prednisone good response; PPR, prednisone poor response). The second word represents day 15 FCM-MRD value (Intermediate = 1–10%; High = 10–100%). CIR, cumulative incidence of relapse.

Supplementary Figure 2 Kaplan-Meyer curves showing the dynamics of day 15 and day 33 MRD. The first word represents the prednisone response (PGR, prednisone good response; PPR, prednisone poor response). The second word represents day 33 MRD value (Low = <0.05%; High = >0.05%). CIR, cumulative incidence of relapse.
