# Supplementary figures and images for: Day 15 and Day 33 Minimal Residual Disease Assessment for Acute Lymphoblastic Leukemia Patients Treated According to the BFM ALL IC 2009 Protocol: Single-Center Experience of 133 Cases
Source: Front Oncol. 2020 Jun 30;10:923. doi: 10.3389/fonc.2020.00923 (PMC7338564; doi:10.3389/fonc.2020.00923)

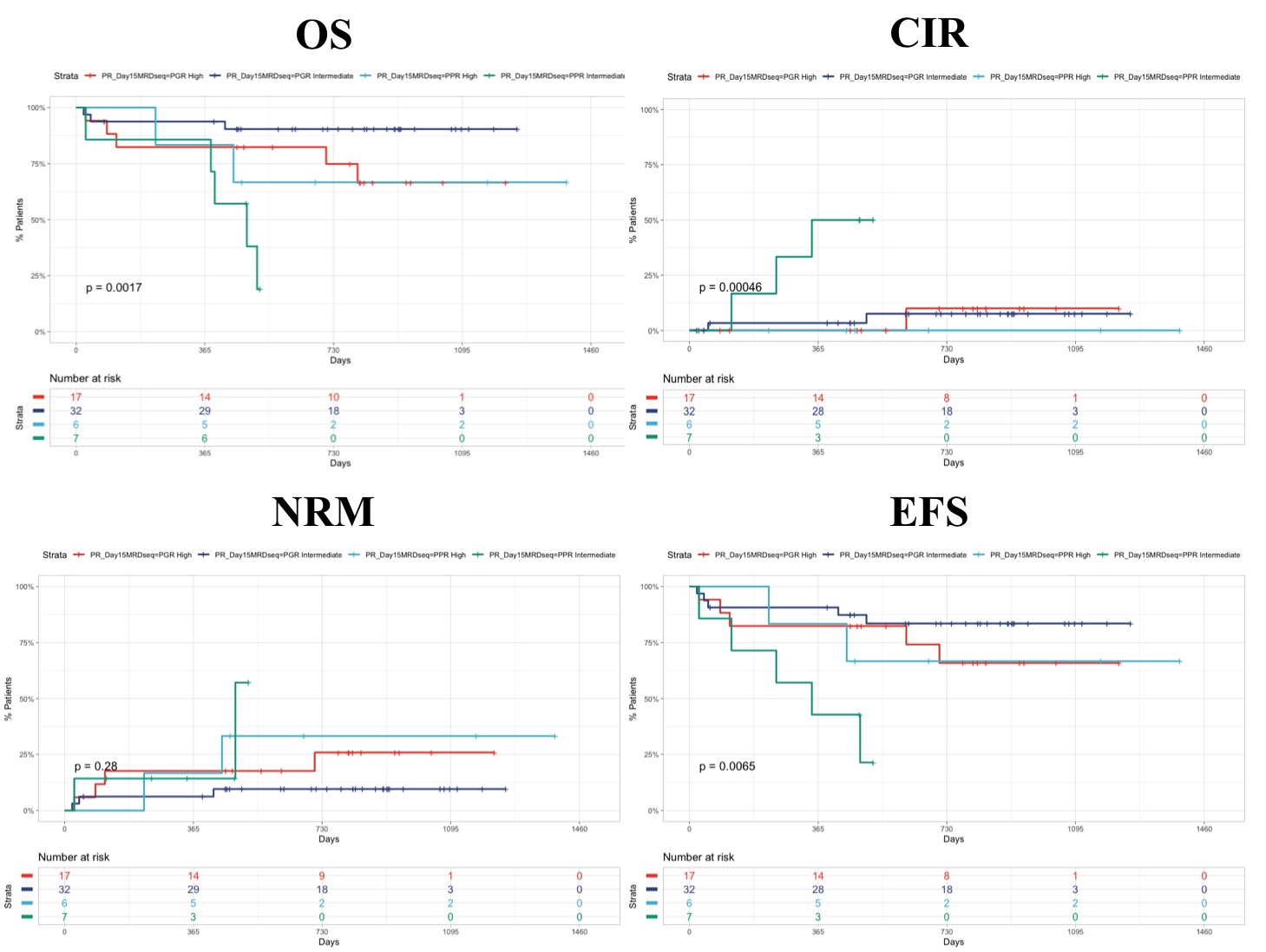

Supplement: Supplementary Figure 1 — Kaplan-Meyer curves showing the dynamics of prednisone response and day 15 FCM-MRD. The first word represents the prednisone response (PGR, prednisone good response; PPR, prednisone poor response). The second word represents day 15 FCM-MRD value (Intermediate = 1–10%; High = 10–100%). CIR, cumulative incidence of relapse. [file Image_1.JPEG]

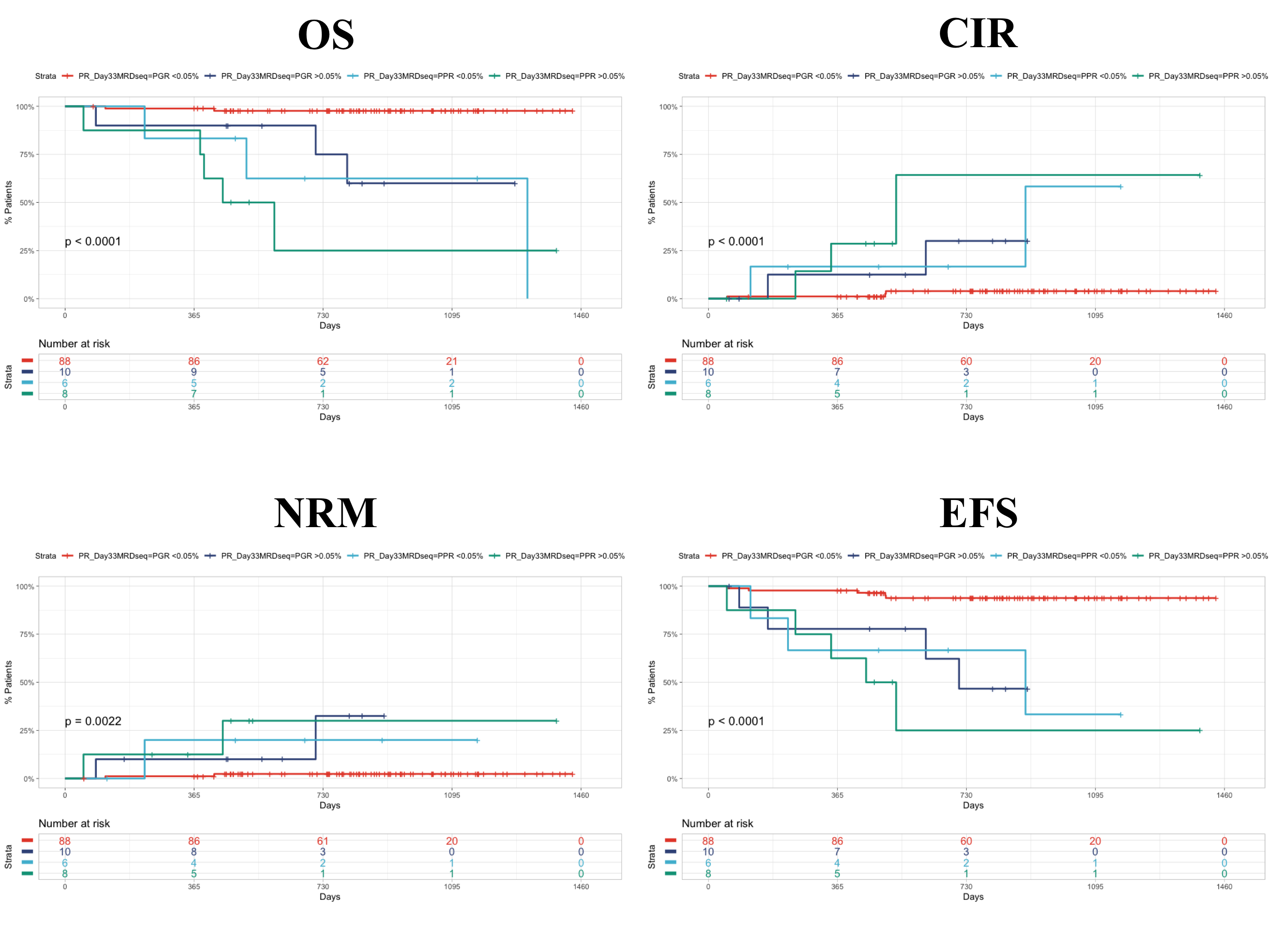

Supplement: Supplementary Figure 2 — Kaplan-Meyer curves showing the dynamics of day 15 and day 33 MRD. The first word represents the prednisone response (PGR, prednisone good response; PPR, prednisone poor response). The second word represents day 33 MRD value (Low = <0.05%; High = >0.05%). CIR, cumulative incidence of relapse. [file Image_2.tiff]
