# Supplementary material for: Day 15 and Day 33 Minimal Residual Disease Assessment for Acute Lymphoblastic Leukemia Patients Treated According to the BFM ALL IC 2009 Protocol: Single-Center Experience of 133 Cases
Source: Front Oncol. 2020 Jun 30;10:923. doi: 10.3389/fonc.2020.00923 (PMC7338564; doi:10.3389/fonc.2020.00923)
Supplement: Supplementary file 3 [file Table_1.docx]

**Supplementary Table 1.** Induction-related death. None of the patients with L2 morphology died before day 33 (Log-rank p value = 0.69); None of the patients with preB immunophenotype died before day 33 (Log-rank p value = 0.27); None of the patients with standard or intermediate risk group died before day 33 (Log-rank p value = 0.16); None of the patients with under 1% blasts at day 33 died before day 33 (Log-rank p value = 0.11).

| **Variable** | **HR** | **Lower 95% CI** | **Upper 95% CI** | **p value** |
| --- | --- | --- | --- | --- |
| Male sex | 0.16 | 0.018 | 1.5 | 0.105 |
| Urban area | 3.2 | 0.36 | 29 | 0.296 |
| Age 10y or more | 4.2 | 0.7 | 25 | 0.118 |
| Leukocytes < 100 x10^9^/L | 0.6 | 0.068 | 5.4 | 0.653 |
| Hb < 7g/dL | 0.86 | 0.096 | 7.7 | 0.89 |
| Platelets < 50 x10^9^/L | 1.6 | 0.27 | 9.6 | 0.607 |
| L2 Morphology | NA | NA | NA | NA |
| T-ALL | 1.3 | 0.14 | 12 | 0.817 |
| preB vs common B | NA | NA | NA | NA |
| Poor Prednisone Response | 1.6 | 0.18 | 14 | 0.683 |
| NonHigh Risk Group | NA | NA | NA | NA |
| Day 15 bone marrow morphologic disease M1 | ref | ref | ref | ref |
| Day 15 bone marrow morphologic disease M2 | 2 | 0.13 | 32 | 0.622 |
| Day 15 bone marrow morphologic disease M3 | 6 | 0.37 | 96 | 0.206 |
| Day 15 FCM-MRD over 1% | NA | NA | NA | NA |
