# Supplementary material for: Day 15 and Day 33 Minimal Residual Disease Assessment for Acute Lymphoblastic Leukemia Patients Treated According to the BFM ALL IC 2009 Protocol: Single-Center Experience of 133 Cases
Source: Front Oncol. 2020 Jun 30;10:923. doi: 10.3389/fonc.2020.00923 (PMC7338564; doi:10.3389/fonc.2020.00923)
Supplement: Supplementary file 4 [file Table_2.docx]

**Supplementary Table 2.** RFS univariate analysis. None of the patients that presented L2 morphology relapsed at follow-up (Log-Rank p value = 0.53).

| **Variable** | **HR** | **Lower 95% CI** | **Upper 95% CI** | **p value** |
| --- | --- | --- | --- | --- |
| Male sex | 2.2 | 0.47 | 10 | 0.316 |
| Urban area | 0.36 | 0.094 | 1.4 | 0.143 |
| Age 10y or more | 4.7 | 1.3 | 17 | **0.017** |
|  |  |  |  |  |
| Leukocytes < 100 x10^9^/L | 0.18 | 0.049 | 0.63 | **0.008** |
| Hb < 7g/dL | 1.5 | 0.39 | 5.9 | 0.54 |
| Platelets < 50 x10^9^/L | 4.6 | 0.97 | 22 | 0.054 |
| L2 Morphology | NA | NA | NA | NA |
| T-ALL | 4.1 | 1.2 | 15 | **0.028** |
| preB vs common B | 3 | 0.61 | 15 | 0.175 |
| Poor Prednisone Response | 10 | 2.9 | 35 | **<0.001** |
| Non-high-Risk Group | 0.16 | 0.04 | 0.61 | **0.007** |
| Day 15 bone marrow morphologic disease M1 | ref | ref | ref | ref |
| Day 15 bone marrow morphologic disease M2 | 1.1 | 0.2 | 6.1 | 0.902 |
| Day 15 bone marrow morphologic disease M3 | 2.6 | 0.29 | 23.4 | 0.397 |
| Day 15 FCM-MRD over 1% | 2.2 | 0.55 | 8.8 | 0.267 |
| Day 33 bone marrow morphologic disease | 9.2 | 2.5 | 33 | **<0.001** |
| Day 33 FCM-MRD over 0.05% | 8.1 | 2.3 | 29 | **0.001** |
