# Supplementary material for: Day 15 and Day 33 Minimal Residual Disease Assessment for Acute Lymphoblastic Leukemia Patients Treated According to the BFM ALL IC 2009 Protocol: Single-Center Experience of 133 Cases
Source: Front Oncol. 2020 Jun 30;10:923. doi: 10.3389/fonc.2020.00923 (PMC7338564; doi:10.3389/fonc.2020.00923)
Supplement: Supplementary file 5 [file Table_3.docx]

**Supplementary Table 3.** RFS multivariate analysis.

| **Variable** | **HR** | **Lower 95% CI** | **Upper 95% CI** | **p value** |
| --- | --- | --- | --- | --- |
| Age 10y or more | 2.92 | 0.793 | 10.7 | 0.107 |
| Leukocytes < 100 x10^9^/L | 0.28 | 0.064 | 1.2 | 0.095 |
| Platelets < 50 x10^9^/L | 3.97 | 0.773 | 20.4 | 0.099 |
| T-ALL | 1.18 | 0.304 | 4.6 | 0.811 |
| Poor Prednisone Response | 4.11 | 0.891 | 19 | 0.07 |
| Day 33 FCM-MRD over 0.05% | 2.56 | 0.507 | 12.9 | 0.255 |
