# Supplementary material for: Day 15 and Day 33 Minimal Residual Disease Assessment for Acute Lymphoblastic Leukemia Patients Treated According to the BFM ALL IC 2009 Protocol: Single-Center Experience of 133 Cases
Source: Front Oncol. 2020 Jun 30;10:923. doi: 10.3389/fonc.2020.00923 (PMC7338564; doi:10.3389/fonc.2020.00923)
Supplement: Supplementary file 6 [file Table_4.docx]

**Supplementary Table 4.** NRM univariate analysis. None of the patients presenting L2 morphology died without having relapsed at follow-up (Log-Rank p value = 0.47); None of the SRG or IRG Risk Group patients died without having relapsed at follow-up (Log-Rank p value < 0.0001); None of the patients that had FCM-MRD under 1% died without having relapsed at follow-up (Log-Rank p value = 0.0011)

| **Variable** | **HR** | **Lower 95% CI** | **Upper 95% CI** | **p value** |
| --- | --- | --- | --- | --- |
| Male sex | 0.22 | 0.07 | 0.7 | **0.01** |
| Urban area | 2.2 | 0.71 | 7 | 0.167 |
| Age 10y or more | 2.6 | 0.93 | 7.1 | 0.068 |
| Leukocytes < 100 x10^9^/L | 0.93 | 0.21 | 4.1 | 0.925 |
| Hb < 7g/dL | 0.24 | 0.032 | 1.9 | 0.172 |
| Platelets < 50 x10^9^/L | 1.7 | 0.61 | 4.8 | 0.311 |
| L2 Morphology | NA | NA | NA | NA |
| T-ALL | 1.3 | 0.38 | 4.8 | 0.648 |
| preB vs common B | 0.3 | 0.039 | 2.3 | 0.247 |
| Poor Prednisone Response | 3.6 | 1.2 | 11 | **0.02** |
| Non-high Risk Group | NA | NA | NA | NA |
| Day 15 bone marrow morphologic disease M1 | ref | ref | ref | ref |
| Day 15 bone marrow morphologic disease M2 | 2 | 0.13 | 33 | 0.615 |
| Day 15 bone marrow morphologic disease M3 | 38 | 4.41 | 335 | **<0.001** |
| Day 15 FCM-MRD over 1% | NA | NA | NA | NA |
| Day 33 bone marrow morphologic disease | 5.7 | 1.1 | 29 | **0.033** |
| Day 33 FCM-MRD over 0.05% | 8.9 | 2 | 40 | **0.005** |
