# Supplementary material for: Day 15 and Day 33 Minimal Residual Disease Assessment for Acute Lymphoblastic Leukemia Patients Treated According to the BFM ALL IC 2009 Protocol: Single-Center Experience of 133 Cases
Source: Front Oncol. 2020 Jun 30;10:923. doi: 10.3389/fonc.2020.00923 (PMC7338564; doi:10.3389/fonc.2020.00923)
Supplement: Supplementary file 7 [file Table_5.docx]

**Supplementary Table 5.** NRM multivariate analysis.

| **Variable** | **HR** | **Lower 95% CI** | **Upper 95% CI** | **p value** |
| --- | --- | --- | --- | --- |
| Male sex | 2.7 | 0.54 | 13 | 0.229 |
| Age 10y or more | 3.8 | 1.05 | 14 | **0.041** |
| Poor Prednisone Response | 4.2 | 1.06 | 16 | **0.04** |
| Day 33 FCM-MRD over 0.05% | 4.5 | 1.1 | 18 | **0.037** |
