# Supplementary material for: Day 15 and Day 33 Minimal Residual Disease Assessment for Acute Lymphoblastic Leukemia Patients Treated According to the BFM ALL IC 2009 Protocol: Single-Center Experience of 133 Cases
Source: Front Oncol. 2020 Jun 30;10:923. doi: 10.3389/fonc.2020.00923 (PMC7338564; doi:10.3389/fonc.2020.00923)
Supplement: Supplementary file 8 [file Table_6.docx]

**Supplementary Table 6.** OS univariate analysis. Only 4 patients presented L2 morphology and none of them died at follow-up (Log-Rank p value = 0.4); None of the standard and intermediate risk patients died at follow-up (Log-Rank p value < 0.0001).

| **Variable** | **HR** | **Lower 95% CI** | **Upper 95% CI** | **p value** |
| --- | --- | --- | --- | --- |
| Male sex | 0.36 | 0.15 | 0.87 | **0.023** |
| Urban area | 1.7 | 0.67 | 4.1 | 0.276 |
| Age 10y or more | 2.3 | 0.95 | 5.4 | 0.065 |
| Leukocytes < 100 x10^9^/L | 0.44 | 0.16 | 1.2 | 0.111 |
| Hb < 7 g/dL | 0.34 | 0.078 | 1.5 | 0.145 |
| Platelets < 50 x10^9^/L | 2.3 | 0.93 | 5.7 | 0.073 |
| L2 Morphology | NA | NA | NA | NA |
| T-ALL | 1.6 | 0.58 | 4.4 | 0.368 |
| preB vs common B | 0.73 | 0.21 | 2.6 | 0.621 |
| Poor Prednisone Response | 6.6 | 2.8 | 16 | **<0.001** |
| Non-high Risk Group | NA | NA | NA | NA |
| Day 15 bone marrow morphologic disease M1 | ref | ref | ref | ref |
| Day 15 bone marrow morphologic disease M2 | 2 | 0.13 | 33 | 0.615 |
| Day 15 bone marrow morphologic disease M3 | 38 | 4.41 | 335 | **<0.001** |
| Day 15 FCM-MRD over 1% | 8.9 | 2 | 41 | **0.005** |
| Day 33 bone marrow morphologic disease | 6.6 | 2 | 22 | **0.002** |
| Day 33 FCM-MRD over 0.05% | 11 | 3.5 | 33 | **<0.001** |
