# Supplementary material for: Day 15 and Day 33 Minimal Residual Disease Assessment for Acute Lymphoblastic Leukemia Patients Treated According to the BFM ALL IC 2009 Protocol: Single-Center Experience of 133 Cases
Source: Front Oncol. 2020 Jun 30;10:923. doi: 10.3389/fonc.2020.00923 (PMC7338564; doi:10.3389/fonc.2020.00923)
Supplement: Supplementary file 9 [file Table_7.docx]

**Supplementary Table 7.** OS multivariate analysis.

| **Variable** | **HR** | **Lower 95% CI** | **Upper 95% CI** | **p value** |
| --- | --- | --- | --- | --- |
| Male sex | 0.51 | 0.16 | 1.6 | 0.251 |
| Age 10y or more | 1.67 | 0.53 | 5.2 | 0.379 |
| Platelets < 50 x10^9^/L | 1.78 | 0.52 | 6.1 | 0.359 |
| Poor Prednisone Response | 7.94 | 2.18 | 29 | **0.002** |
| Day 33 FCM-MRD over 0.05% | 3.65 | 1.03 | 12.9 | **0.045** |
