# Supplementary material for: Day 15 and Day 33 Minimal Residual Disease Assessment for Acute Lymphoblastic Leukemia Patients Treated According to the BFM ALL IC 2009 Protocol: Single-Center Experience of 133 Cases
Source: Front Oncol. 2020 Jun 30;10:923. doi: 10.3389/fonc.2020.00923 (PMC7338564; doi:10.3389/fonc.2020.00923)
Supplement: Supplementary file 10 [file Table_8.docx]

**Supplementary Table 8.** EFS univariate analysis. None of the patients with L2 morphology presented any event at follow-up (Log-rank p value = 0.34).

| **Variable** | **HR** | **Lower 95% CI** | **Upper 95% CI** | **p value** |
| --- | --- | --- | --- | --- |
| Male sex | 0.54 | 0.25 | 1.2 | 0.129 |
| Urban area | 1.1 | 0.48 | 2.3 | 0.887 |
| Age 10y or more | 3.3 | 1.5 | 7.2 | **0.003** |
| Leukocytes < 100 x10^9^/L | 0.42 | 0.17 | 1.1 | 0.067 |
| Hb < 7g/dL | 0.68 | 0.23 | 2 | 0.473 |
| Platelets < 50 x10^9^/L | 2.4 | 1 | 5.6 | **0.038** |
| L2 Morphology | NA | NA | NA | NA |
| T-ALL | 2.3 | 0.95 | 5.5 | 0.064 |
| preB vs common B | 0.91 | 0.3 | 2.8 | 0.871 |
| Poor Prednisone Response | 5.7 | 2.5 | 13 | **<0.001** |
| Non-high Risk Group | 0.066 | 0.19 | 0.22 | **<0.001** |
| Day 15 bone marrow morphologic disease M1 | ref | ref | ref | ref |
| Day 15 bone marrow morphologic disease M2 | 1.3 | 0.31 | 5.4 | 0.719 |
| Day 15 bone marrow morphologic disease M3 | 10.6 | 3.20 | 35.4 | **<0.001** |
| Day 15 FCM-MRD over 1% | 5.8 | 1.7 | 20 | **0.005** |
| Day 33 bone marrow morphologic disease | 7.6 | 2.8 | 21 | **<0.001** |
| Day 33 FCM-MRD over 0.05% | 8.6 | 3.3 | 23 | **<0.001** |
