# Supplementary material for: Day 15 and Day 33 Minimal Residual Disease Assessment for Acute Lymphoblastic Leukemia Patients Treated According to the BFM ALL IC 2009 Protocol: Single-Center Experience of 133 Cases
Source: Front Oncol. 2020 Jun 30;10:923. doi: 10.3389/fonc.2020.00923 (PMC7338564; doi:10.3389/fonc.2020.00923)
Supplement: Supplementary file 11 [file Table_9.docx]

**Supplementary Table 9**. EFS multivariate analysis.

| **Variable** | **HR** | **Lower 95% CI** | **Upper 95% CI** | **p value** |
| --- | --- | --- | --- | --- |
| Age 10y or more | 3.04 | 1.13 | 8.2 | **0.028** |
| Leukocytes < 100 x10^9^/L | 0.57 | 0.18 | 1.8 | 0.334 |
| Platelets < 50 x10^9^/L | 1.63 | 0.55 | 4.8 | 0.375 |
| T-ALL | 1.02 | 0.35 | 3 | 0.965 |
| Poor Prednisone Response | 3.51 | 1.12 | 11 | **0.031** |
| Day 33 FCM-MRD over 0.05% | 3.62 | 1.15 | 11.4 | **0.028** |
